# Metabolic Stress Alters Antioxidant Systems, Suppresses the Adiponectin Receptor 1 and Induces Alzheimer’s Like Pathology in Mice Brain

**DOI:** 10.3390/cells9010249

**Published:** 2020-01-19

**Authors:** Jong Ryeal Hahm, Myeung Hoon Jo, Rahat Ullah, Min Woo Kim, Myeong Ok Kim

**Affiliations:** 1 Division of Endocrinology and Metabolism, Department of Internal Medicine, Gyeongsang National University Hospital and Institute of Health Sciences and Department of Internal Medicine, College of Medicine, Gyeongsang National University, Jinju 52828, Korea; jrhahm@gnu.ac.kr; 2 Division of Life Sciences and Applied Life Science (BK 21plus), College of Natural Science, Gyeongsang National University, Jinju 52828, Korea; audgns1217@gnu.ac.kr (M.H.J.); rahatullah1414@gnu.ac.kr (R.U.); mwkim0322@gnu.ac.kr (M.W.K.)

**Keywords:** obesity, oxidative stress, neuroinflammation, insulin resistance, amyloid-beta, neurodegeneration

## Abstract

Oxidative stress and insulin resistance play major roles in numerous neurodegenerative diseases, including Alzheimer’s disease (AD). A high-fat diet induces obesity-associated oxidative stress, neuronal insulin resistance, microglial activation, and neuroinflammation, which are considered important risk factors for neurodegeneration. Obesity-related metabolic dysfunction is a risk factor for cognitive decline. The present study aimed to elucidate whether chronic consumption of a high-fat diet (HFD; 24 weeks) can induce insulin resistance, neuroinflammation, and amyloid beta (Aβ) deposition in mouse brains. Male C57BL/6N mice were used for a high-fat diet (HFD)-induced pre-clinical model of obesity. The protein expression levels were examined via Western blot, immunofluorescence, and the behavior analysis was performed using the Morris water maze test. To obtain metabolic parameters, insulin sensitivity and glucose tolerance tests were performed. We found that metabolic perturbations from the chronic consumption of HFD elevated neuronal oxidative stress and insulin resistance through adiponectin receptor (AdipoR1) suppression in HFD-fed mice. Similarly, our in vitro results also indicated that knockdown of AdipoR1 in the embryonic mouse hippocampal cell line mHippoE-14 leads to increased oxidative stress in neurons. In addition, HFD markedly increased neuroinflammatory markers’ glial activation in the cortex and hippocampus regions of HFD mouse brains. More importantly, we observed that AdipoR1 suppression increased the amyloidogenic pathway both in vivo and in vitro. Furthermore, deregulated synaptic proteins and behavioral deficits were observed in the HFD mouse brains. Taken together, our findings suggest that excessive consumption of an HFD has a profound impact on brain function, which involves the acceleration of cognitive impairment due to increased obesity-associated oxidative stress, insulin resistance, and neuroinflammation, which ultimately may cause early onset of Alzheimer’s pathology via the suppression of AdipoR1 signaling in the brain.

## 1. Introduction

Oxidative stress plays a key role in several neurological complications, including Alzheimer’s disease (AD). Studies reported that diet-induced oxidative stress leads to metabolic syndrome due to loss of balance between the ingested diet and energy consumption or dysfunction of adipose tissue [1]. Obesity, the incidence of which is increasing globally, has received increasing attention, and is considered a key driving force in metabolic syndrome [2]. At present, 1.9 billion people are obese or overweight and thus have a reduced life expectancy [3]. Indeed, epidemiologic studies in animal models have shown strong associations among obesity, metabolic dysfunction, and neurodegeneration [4]. A previous study found that individuals who consume high-calorie diets are 1.5-times more prone to develop AD than individuals who consume low-calorie diets [5,6]. Obesity causes damage to several tissues, including brain and non-brain (heart, pancreas, skeletal muscle, kidney, and liver) tissues [7]. Obesity caused dysfunction of adipose tissue resulting in abnormal levels of several circulating adipokines, including adiponectin [8]. In the brain, adiponectin receptors, notably AdipoR1, are widely expressed, including cortex and hippocampus [9]. They have a number of diversified neuroprotective actions [8], and also the regulation of food intake and energy expenditure through AdipoR1-mediated AMP-activated protein kinase (AMPK) signaling [10]. Of note, AdipoR1-mediated signaling appears to be neuroprotective against metabolic insults, including HFD in the brain [11]. However, obesity reduced circulating levels of adiponectin [8]. In this regard, adiponectin-deficient mice showed decreased AMPK phosphorylation, food intake, and increased energy expenditure, thus exhibiting resistance to HFD-induced obesity [10]. Similarly, others reported that aged adiponectin-knockout mice induce an AD-like phenotype in experimental mice [8]. Specifically, the silencing of adipoR1 has also been reported in aggravating brain pathology in AD-mice [12]. Furthermore, suppression of adiponectin receptor 1 (AdipoR1) via gene-therapy also induces metabolic dysfunction and the AD-like phenotype [13]. Collectively, these studies demonstrated a strong association between adiponectin receptor 1 (AdipoR1) and the development of AD pathogenesis.

Studies have shown that chronic adiponectin deficiency leads to cerebral insulin resistance in aged mice [14]. Brain insulin signaling plays a key role in learning and memory [15]. HFD-induced disruption of the brain’s insulin signaling has been reported to increase the risk of developing cognitive impairment and dementia [16,17,18]. Mounting evidence links diabetes and obesity to Alzheimer’s disease (AD), and as a result, AD can be regarded as type 3 diabetes or insulin-resistant brain disease [19,20,21]. Obesity is a risk factor and a common predating symptom for both metabolic and neurodegenerative diseases; therefore, the control of mid-life obesity might be a therapeutic strategy for reducing cognitive decline. The results from clinical studies suggest that obesity-induced inflammation in adipose tissue is an important risk factor for cognitive impairment in elderly populations [22,23]. It has been reported that the onset of obesity, neuroinflammation, and microglial activation is increased by the consumption of a diet rich in saturated fat [24]. In obesity, defects in adipocyte or adipose tissue inflammation cause neuronal dysfunction by producing cytokines, adipokines, and chemokines, and these peripheral inflammatory mediators, along with microglial inflammatory mediators, enhance central nervous system (CNS) inflammation [25,26]. In obese individuals, TNF-α is overexpressed in adipose tissue and activates neuronal cytokine receptors (e.g., TNF-α receptor), and this effect causes aberrant activation of stress kinases that phosphorylate insulin receptor substrate 1 (IRS-1) at serine residues, while inhibiting tyrosine phosphorylation of IRS-1, leading to insulin resistance [27,28,29].

Studies have shown a strong association between neuroinflammation and brain insulin resistance leading to amyloid pathology [30]. Similarly, accumulative lines of evidence demonstrate that the consumption of a high-fat diet (HFD) increases amyloid-beta protein (Aβ) deposition by inhibiting Aβ degradation and clearance in amyloid precursor protein (APP) transgenic mice [31,32,33,34], which supports the notion that mid-life obesity is a risk factor for neurodegenerative disorders, such as AD and PD [35,36]. Other research groups have shown that the consumption of a high-fat diet promotes Aβ production [31], leading to hippocampus-dependent spatial memory impairment, deficits in synaptic plasticity and reduced neurogenesis in the dentate gyrus, and these findings have been observed in both rodent models of diet-induced obesity (DIO) and transgenic models of obesity [37,38,39].

The aim of the present study was to establish a connection between the associated effects of HFD-induced metabolic stress on the antioxidant system, brain insulin signaling, AdipoR1-associated signaling, and the early onset of pathological changes associated with Alzheimer’s disease in the pre-clinical model of obesity.

## 2. Materials and Methods

### 2.1. Chemicals

MTT (3-(4,5-dimethylthiazol-2-yl)-2,5-diphenyltetrazolium bromide, dimethyl sulfoxide (DMSO), and d-(+)-glucose solution were purchased from Sigma Aldrich Chemicals Company (St. Louis, MO, USA).

### 2.2. Animals and Diets

Male, wild-type C57BL/6N mice (aged eight weeks, weighing 25–30 g) were purchased from Samtako Bio (Osan, Korea). All experimental mice acclimatized for one week under standard laboratory conditions in a temperature (23 ± 2 °C)- and humidity (60 ± 15%)-controlled facility with a 12 h light/12 h dark cycle. The mice were randomly assigned to one of two groups (n = 12 mice/group): Group 1, the control (C) mice were fed ad libitum a standard normal chow diet (NCD) containing proteins (20% kcal), carbohydrates (70% kcal), and fats (10% kcal), per dry weight (Cat. #D12450B, Open Source Diet, Research Diets, Inc., New Brunswick, NJ, USA); and Group 2, the mice were fed with HFD containing proteins (20% kcal), carbohydrates (20% kcal), and fats (60% kcal), per dry weight (Cat. #D12492, Open Source Diet, Research Diets, Inc., New Brunswick, NJ, USA) for six months (24 weeks). A behavioral analysis of the mice was then conducted. The mice were sacrificed for protein expression and immunohistochemical analysis. All the experimental procedures were carried out in accordance with the local ethical committee for animal research of the Department of Biology, Division of Applied Life Sciences, Gyeongsang National University South Korea, who approved the experimental procedures (Approval ID: 125).

### 2.3. Behavioral Analysis: Morris Water Maze (MWM) and Y-Maze Test

The MWM test was used for the evaluation of spatial learning and memory [40,41]. The experimental apparatus consisted of a black circular plastic pool (diameter = 100 cm) located in the center of the room and filled with water (temperature 23 ± 1 °C, depth 40 cm). In addition, the pool was painted with an opaque, nontoxic white tempera paint to obscure the location of a black round platform (10 × 10 cm) located 1–2 cm below the water surface and was divided into four equal quadrants (NE, corresponding to the target quadrant, SE, NW, and SW). In brief, during the training session, each individual mouse was first trained in the experimental swimming pool apparatus and was then subjected to four swimming trials per day (each with a different starting position) with a 20 min intertrial interval for four consecutive days. An automated video tracking system (SMART, Panlab Harvard Apparatus Bioscience Company, Holliston, MA, USA) was used to record the time that each mouse needed to find the hidden platform (escape latency) and the distance traveled (path length). In the probe test, the platform was removed. The time that each mouse spent in the target quadrant for 60 s, and the number of platform crossings was measured and analyzed.

In each session of the Y-maze test (length = 20 cm, height = 20 cm, width = 20 cm from top and bottom), each mouse was placed in the center and allowed to move in the apparatus for 3–8 min freely. The series of arm entries was visually observed, and a spontaneous alteration was defined as successive entries into the three arms in overlapping triplet sets. The alteration behavior percentage (%) was calculated as [successive triplet sets (consecutive entries into three different arms)/total number of arms entries − 2] × 100. A higher percentage of spontaneous alternations was considered to indicate enhanced cognitive performance.

### 2.4. Insulin Sensitivity and Glucose Tolerance Tests

Insulin sensitivity and glucose tolerance tests were conducted as previously reported [42]. The mice (n = 12 mice/group) were fasted overnight (16 h) and then subjected to the glucose tolerance test (GTT) and the insulin tolerance test (ITT). Each mouse received D(+) glucose (1–2 g/kg body weight) through intraperitoneal injection, and a blood sample was drawn from the tail vein of the mouse. The serum glucose levels were then measured using a glucometer (OneTouch UltraMini, LifeScan, Milpitas, CA, USA). An insulin tolerance test (ITT) was conducted. Each mouse received an intraperitoneal injection of insulin (0.75 units/kg in 0.1 mL of 0.9% normal saline; Humulin-R, Eli Lilly and Company, Indianapolis, IN, USA), and blood samples were drawn from the tail vein of the mouse at baseline (time = 0 min) and 15, 30, 60, and 120 min after injection. The serum glucose levels were then measured using a glucometer (OneTouch UltraMini; LifeScan, Milpitas, CA, USA).

### 2.5. Extraction of Murine Brain Proteins

The mice were sacrificed after the behavioral analyses. Briefly, the brains were carefully removed and dissected. Both the cortex and hippocampus region of the murine brain were homogenized in 0.2 M PBS with a phosphatase inhibitor and protease inhibitor cocktail using the Pro-Prep protein extraction solution (PRO-PREP) according to the manufacturer’s instructions (iNtRON Biotechnology, Inc., Seongnam, South Korea). The samples were then centrifuged at 10,000× *g* and 4 °C for 25 min. The supernatants were collected and stored at −80 °C.

### 2.6. Western Blotting Analysis

Western blot analysis was conducted as previously described to measure the expression levels of different proteins [43,44]. Briefly, the optical densities (O.D) of the proteins in brain homogenates were measured using a Bio-Rad protein assay kit (Bio-Rad Laboratories, CA, USA). The brain homogenates (20−30 μg per sample) were fractionated by sodium dodecyl sulfate polyacrylamide gel electrophoresis (SDS-PAGE) and then transferred to a polyvinylidene difluoride (PVDF) membrane. To detect the molecular weights of the desired proteins, restained protein ladders (GangNam-STAINTM, iNtRON Biotechnology, Inc., Kyungki-Do, Republic of Korea) that covered a broad range of molecular weights were loaded. After protein transfer, the PVDF membrane was blocked with 5% (*w*/*v*) skim milk or bovine serum albumin (BSA) to reduce nonspecific binding and incubated with the primary antibody (1:1000 dilution) at 4 °C overnight and then, with horseradish peroxidase-conjugated secondary antibody. The proteins were detected using a chemiluminescence-based detection kit (ECL kit; Amersham, Japan) according to the manufacturer’s instructions. The scanned X-ray films were processed for densitometry analysis to analyze the density values of the bands in terms of arbitrary units (A.U.) using the ImageJ software (Version 1.8.0_112).

### 2.7. Cell Culturing

Embryonic Mouse Hippocampal Cell Line mHippoE-14 (Cat # CLU198 E14, Cedar Lane, Ontario Canada) cells were cultured according to supplies instructions in Dulbecco’s modified Eagle media without sodium pyruvate (Gibco Fisher Scientific, Leicestershire, UK) supplemented with 10% Fetal bovine serum (Gibco Fisher Scientific, Leicestershire, Leicestershire, UK) and 5% Penicillin-Streptomycin (10,000 U/mL) (Gibco Fisher Scientific, Leicestershire, Leicestershire, UK). Cells were revived and cultured till confluency in a 37 °C cell culturing incubator with 5% carbon dioxide.

### 2.8. Cell Transfection

Cell seeded and grown in Nunc™ Cell-Culture Treated culturing six-well plates till it reached almost 70% confluency. AdipoR1 gene was knocked down by Sure Silencing Mouse ApidoR1 shRNA plasmid (Qiagen, Hilden-Düsseldorf, Germany). The plasmid was transfected using lipid-mediated transfection using Lipofectamine 3000 reagent (Thermo-Fisher Scientific, Waltham, MA, USA) according to the manufacturer’s protocols. A quantity of 3.5 µg of plasmid was transfected per well. Transfection mixtures were made according to the protocol in Opti-MEM reduced serum media (Gibco Fisher Scientific, Leicestershire, UK). The transfection mixture was added drop by drop in the wells, and cells were incubated for 2 h for proper absorption of the transfection mixture containing AdipoR1 shRNA plasmid. Cells were further cultured in normal cell culture media for 72 h. Cells were later harvested for protein quantification by Western blot analysis.

### 2.9. Detection of Oxidative Stress (In Vivo and In Vitro)

The level of reactive oxygen species (ROS) was assayed using a previously reported method with slight modifications [45] based on the oxidation of 2,7 dicholorodihydrofluoresceindiacetate (DCFH-DA) to 2,7-dichlorodihydrofluorescein (DCF). The brain homogenates were diluted in ice-cold Locke’s buffer at 1:20 to obtain a concentration of 5 mg tissue/mL. The reaction mixture (1 mL), which included Locke’s buffer at pH 7.4, 0.2 mL of brain homogenate and 10 mL of DCFH-DA (5 mM), was incubated for 30 min at room temperature to allow the conversion of DCFH-DA to DCF. The fluorescence was detected using a spectrofluorometer (Promega Bioscience, CA, USA) with excitation and emission wavelengths of 484 and 530 nm, respectively. The ROS content is expressed as pmol DCF formed/min/mg protein. In addition, ROS quantification/level was also performed in embryonic mouse hippocampal cell line mHippoE-14, as discussed for the in vivo experiment.

### 2.10. Assessment of Lipid Peroxidation (In Vivo and In Vitro)

Lipid peroxidation (LPO) quantification assays were conducted to assess oxidative stress [46]. Briefly, the levels of free malondialdehyde (MDA), a marker of LPO, in brain homogenates, as well as in the embryonic mouse hippocampal cell line mHippoE-14, were measured using a lipid peroxidation (MDA) colorimetric/fluorometric assay kit (Bio Vision, Milpitas, CA, USA, Cat #K739-100), according to the manufacturer’s recommended protocol.

### 2.11. Glutathione Assays

The total glutathione (GSH; in reduced states) and ratio of GSH/GSSG (oxidized glutathione (GSSG) levels were determined using a glutathione assay kit (BioVision Incorporated, 155 S. Milpitas Boulevard, Milpitas, CA, USA) and a fluorometric assay kit, according to the manufacturer’s instructions.

### 2.12. Immunofluorescence

The murine brains were fixed through transcranial perfusion with 4% ice-cold paraformaldehyde, as previously reported [47], for 72 h in 4% paraformaldehyde and then, with 20% sucrose for an additional 72 h at 4 °C. The brains were mounted using the optimal cutting temperature (OCT) compound (Tissue-Teks O.C.T. Compound Medium, Sakura Finetek USA, Inc., Torrance, CA, USA), frozen in liquid nitrogen, and sectioned (14 µm) in the coronal plane using a CM 3050C cryostat (Leica, Wetzlar, Germany). For immunofluorescence analysis, the slides were washed twice with 1× PBS, incubated with proteinase K solution and blocked with normal goat/rabbit serum (5% normal goat/rabbit serum, 0.3% Triton X-100, and PBS). The brain slices were incubated with primary antibodies (1:100 in PBS) overnight at 4 °C and then with fluorescence-conjugated (FITC and TRITC from Santa Cruz Biotechnology (Santa Cruz, CA, USA), Cell Signaling Technology ((Danvers, MA, USA), and Abcam, secondary antibodies (in PBS) at room temperature. The nuclei were stained with DAPI (4’,6-diamidino-2-phenylindole). The slides were mounted with glass coverslips, and images were obtained using a confocal microscope (FluoView FV1000; Olympus, Tokyo, Japan).

### 2.13. Antibodies

The following primary antibodies were used in the Western blot analysis: rabbit-sourced antibodies, anti-APP, anti-AdipoR1, anti-pAMPK((Thr172), anti-AMPK, anti-phospho insulin receptor substrate (pIRS-1;ser 636), anti-pPI3K, anti-p-AKT (ser473), and anti-AKT (all from Cell Signaling Technology, Beverly, MA, USA); and mouse-sourced antibodies, anti-beta-amyloid-cleaving enzyme (BACE-1), anti-nuclear factor kappa B (NFkB), anti-phospho-c-Jun n-terminal kinase (p-JNK), anti-tumor necrosis factor-alpha (TNFα), anti-interleukin 1 βeta (IL1β), anti-ionized calcium binding adapter molecule 1 (Iba-1), anti-Glial fibrillary acidic protein (GFAP), anti-amyloid beta (Aβ;B4), anti-postsynaptic density proteins (PSD-95), anti- synaptosomal-associated protein 23 (SNAP23), anti-nuclear factor erythroid-2-related factor 2 (Nrf2), anti-hemeoxygenase 1(HO1) and anti-βeta-actin (β-actin) (all from Santa Cruz, Biotechnology, CA, USA).

### 2.14. Thioflavin S Staining of Aβ Plaques

Aβ plaques were analyzed through thioflavin S staining [12]. Briefly, brain sections were washed in 0.01 M PBS for 10 min and stained in a Coplin jar containing fresh 1% thioflavin S (dissolved in distilled water; Sigma Chemical Co., St. Louis, MO, USA) for 10 min at room temperature. The sections were then incubated in 70% ethanol for 5 min, rinsed twice in water, and stained with propidium iodide (PI) (Invitrogen, Carlsbad, CA, USA), and glass coverslips were then added. The green fluorescence of thioflavin S was observed using a confocal microscope (FluoView FV1000; Olympus, Tokyo, Japan). For quantitative analysis, the percentage of plaque area per number of plaques was calculated using the ImageJ software.

### 2.15. Data and Statistical Analysis

All the experiments were performed in triplicate, and the data are presented as the means ± standard errors of the mean (SEMs). All the Western blot bands in the original X-ray films were scanned, and ImageJ software was used to quantitatively evaluate the Western blot, immunofluorescence (IF) and immunohistology results. The density values are expressed as the means ± SEMs. One-way ANOVA followed by Student’s *t*-test (nonparametric Mann−Whitney and Wilcoxon tests) was used to assess the significance of the differences between the HFD and control groups. Prism 7 software (GraphPad Software, Inc., San Diego, CA, USA) was used for the calculations and to prepare the graphs. *p*-values < 0.05 were considered statistically significant and the significant results are shown as follows: * *p* ≤ 0.05, ** *p* ≤ 0.01, and *** *p* ≤ 0.001.

## 3. Results

### 3.1. HFD-Induced Obesity Deregulates Metabolic Parameters in Mice

**Figure 1:**
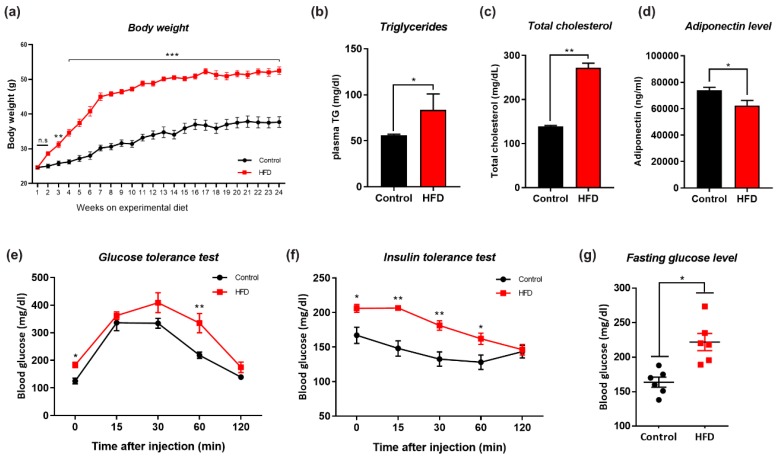
Effects of high fat diet (HFD) on metabolic parameters. Changes in (**a**) Body weight, (**b**) Plasma triglyceride levels, (**c**) Plasma total cholesterol level, (**d**) Plasma adiponectin level, (**e**) Glucose tolerance test, (GTT), (**f**) Insulin tolerance test, (ITT), (**g**) Fasting glucose between normal chow diet (NCD) and HFD fed experimental mice groups. (n = 12 mice/group). Data are presented as mean ± SEM and were analyzed using one-way ANOVAs between NCD and HFD mice groups. Significance = * *p* < 0.05, ** *p* < 0.01, *** *p* < 0.001.

Eight-week-old male mice were divided into two groups (n = 12/group): the mice belonging to one of the groups were fed the NCD for 24 weeks, and the other mice were fed the HFD for 24 weeks to induce obesity. The body weights of the mice and other key parameters related to obesity and insulin resistance were measured during the 24-week feeding period. The control mice, which were fed the NCD, exhibited a constant body weight gain over the 24-week period, whereas the HFD-fed mice showed a significantly higher body weight gain and increased weight gains in peripheral organs, including the liver; however, we observed no marked changes in the kidney and spleen (Figure 1a, Figure S1c). We also found that HFD-fed mice showed significantly increased plasma triglyceride, cholesterol, and significantly lower adiponectin levels compared with their respective control mice (Figure 1b–d). Other studies have shown that high-fat-diet-induced obesity in mice exhibited lower glucose and insulin tolerance versus normal-diet mice [48]. Similarly, the HFD-fed mice showed a significant decrease in insulin sensitivity, as demonstrated by the GTT and ITT results (Figure 1e,f). Interestingly, the NCD (control diet) alone was unable to induce glucose intolerance (Figure 1g). These findings indicate that the chronic consumption of HFD caused increased body weight gains and significant metabolic perturbations that have profound impacts on insulin sensitivity, as reflected by the impaired GTT and ITT results. 

### 3.2. HFD Induces Oxidative-Stress-Mediated Brain Insulin Resistance by Impairing AdipoR1/P-AMPK Signaling Both In Vivo and In Vitro

**Figure 2:**
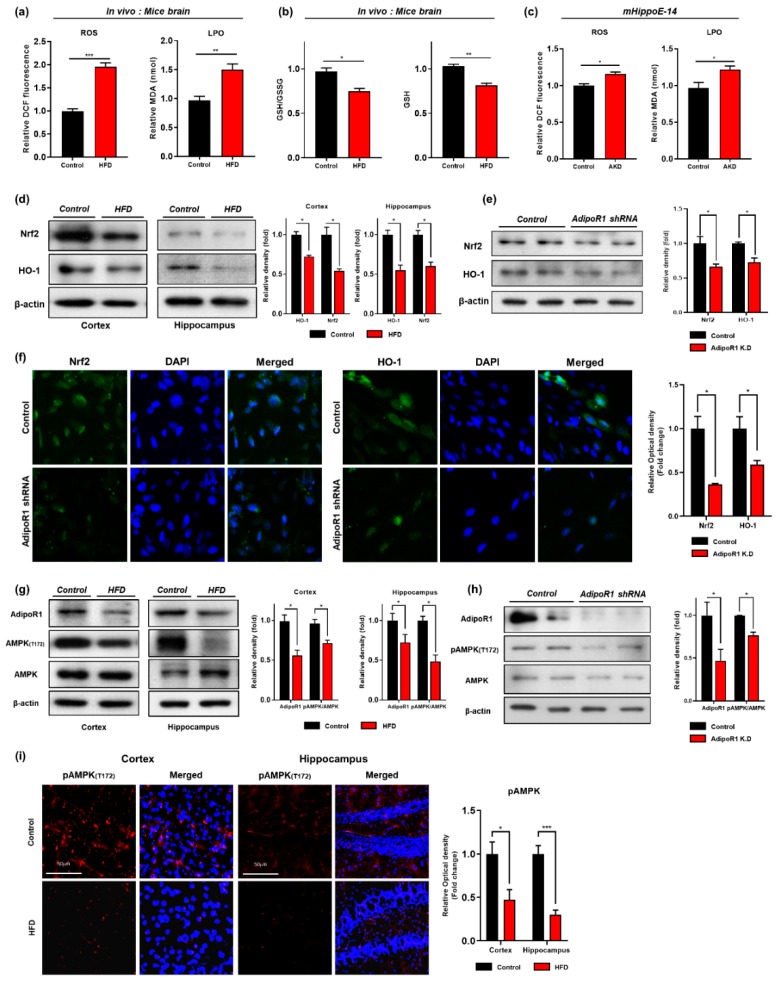
HFD induced Oxidative Stress impaired AdipoR1 signaling pathway in both in vivo and in vitro (**a**) Representative histogram of reactive oxygen species (ROS) and lipid peroxidation (LPO) levels (assay carried out in triplicates) (**b**) and glutathione (GSH; in reduced state) levels (nmol/mg protein) along with GSH/GSSG ratio (GSSG; glutathione in oxidized state) of normal chow and high fed diet mice brains (n = 12 mice/group) (**c**) The histogram showing the results of reactive oxygen species (ROS) and lipid peroxidation (LPO) levels in control and AdipoR1 knockdown in the embryonic mouse hippocampal cell line mHippoE-14 (adipoR1 shRNA) (**d**,**e**) Shows the western blot analysis of nuclear factor-2 erythroid-2 (Nrf-2) and hemeoxygenase-1 (HO-1) along with respective histograms in HFD-fed mice brains and AdipoR1 knockdown embryonic mouse hippocampal cell line mHippoE-14(adipoR1 shRNA) (**f**) Immunofluorescence staining images of Nrf-2 and HO-1 in the control and AdipoR1 knockdown embryonic mouse hippocampal cell line mHippoE-14(adipoR1 shRNA) (**g**,**h**) Shows the western blot analysis of AdipoR1 and its downstream p-AMPK (T172) and total AMPK in both in vivo and in vitro (**i**) Immunofluorescence imaging of phosphorylated AMPK in the cortex and hippocampus region of control and HFD mice brain along with their histograms. β-Actin was used as loading control; n = 12 mice/group. Data are presented as mean ± SEM. Data for immunofluorescence and western experiment are compared using Student *t*-test and other data are compared using one-way ANOVAs. Significance = * *p* < 0.05, ** *p* < 0.01, *** *p* < 0.001.

It has been demonstrated that consumption of a high-calorie diet intake enhanced oxidative stress in several tissues, including brain tissue [34,49]. Similarly, others also demonstrated that consumption of HFD significantly reduced the protein expression level of hemeoxygenase-1 (HO-1) and nuclear factor-2 erythroid-2 (Nrf-2) in HFD–fed mice brains [28]. In order to determine whether consumption of the HFD for 24 weeks mediate oxidative stress, for this purpose, we quantified oxidative stress in terms of ROS/LPO and GSH/GSSG assays both in vitro and in vivo. Our results of ROS and LPO assays indicated that chronic consumption of HFD significantly increased ROS/LPO level in mice brain (Figure 2a). Similarly, we also examined the glutathione (GSH and GSSG) levels and found that consumption of the HFD for 24 weeks significantly reduced the cellular GSH content and the GSH/GSSG ratio in the mouse brains compared with the levels found in the NCD-fed mouse brains (Figure 2b). Moreover, our Western blot results showed a significant reduction in the protein expression level of Nrf-2 and HO-1 in both cortex and hippocampus regions of HFD-fed mice brain (Figure 2d). To ascertain these results of oxidative stress in relation to the possible involvement of AdipoR1. We knockdown adipoR1 in embryonic mouse hippocampal cell line mHippoE-14 by using by Sure Silencing Mouse ApidoR1 shRNA plasmid. Similar to our In vivo findings, we found that adipoR1 knockdown also increased ROS/LPO level and reduced expression of Nrf-2/HO-1 proteins levels, as indicated by immunoblot analysis (Figure 2c,e). Furthermore, our immunofluorescence results also showed significant reductions in the immunoreactivity of Nrf-2/HO-1 protein levels in AdipoR1 knockdown cells ((Figure 2f). From these results, we can conclude that the chronic consumption of HFD-induced oxidative stress by down-regulating the antioxidant defense systems via adipoR1 in HFD-fed mice brains.

Previous studies have reported that, under physiological conditions, adiponectin (APN), through phosphorylation of adiponectin receptor 1 (AdipoR1), activates AMPK, and regulates insulin signaling in the brain [2,50]. However, HFD-induced obesity results in peripheral metabolic changes and significant reductions in AMPK levels in the brain [12,51]. In the current study, we observed that both adipoR1 and phospho-AMPK (Thr172) are downregulated in the HFD-fed mice (both in cortex and hippocampus) compared with their control age-matched littermates via both immunoblot and immunofluorescences analysis (Figure 2h,j). Similarly, these findings were then further strengthened in vitro in which AdipoR1 knockdown downregulates its downstream p-AMPK (T172) supporting our in vivo results (Figure 2i).

**Figure 3:**
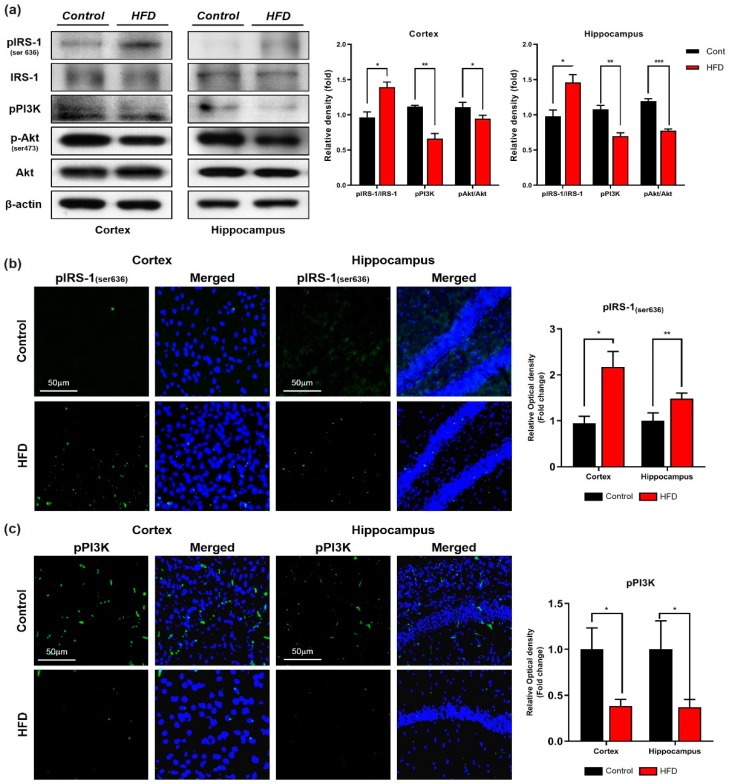
Chronic HFD reduced insulin sensitivity in HFD mice brains. (**a**) Immunoblot representation of insulin signaling pathway markers, phospho-insulin receptor substrate 1 (pIRS-1; 636), phospho-inositol 3-kinase (p-PI3K), and phospho Akt (p-Akt) along with their respective histograms. β-actin protein was used as loading control. (**b**,**c**) Confocal images of insulin signaling markers pIRS and pPI3K in both cortex and hippocampus of control and HFD mice brain. Data are presented as mean ± SEM. Data for immunofluorescence and western blot experiments are compared using Students *t*-test, and other data are compared using one-way ANOVAs. Data are compared using the unpaired Student *t*-test. Significance = * *p* < 0.05, ** *p* < 0.01, *** *p* < 0.001.

Furthermore, other studies have reported that obesity increased central insulin resistance in overweight subjects in comparison with lean subjects [52]. Therefore, to further evaluate the molecular mechanism underlying the HFD-induced brain insulin resistance, we investigated the insulin signaling pathway through Western blotting and immunostaining analyses. Our immunoblot and immunofluorescence results revealed that the expression and immunoreactivity of IRS-1 phosphorylated at serine residue 616 was significantly increased in the cortex and hippocampus of the HFD-fed mice in comparison with the control mice (Figure 3a,b), and this increase was followed by decreases in the levels of phospho-PI3K and phospho-Akt in both the cortex and hippocampus region of the HFD-mice group (Figure 3a,c). Taken together, these findings indicated that HFD-induced oxidative stress mediates neuronal insulin resistance by impairing adipoR1/p-AMPK signaling both in vivo and in vitro.

### 3.3. Chronic HFD Consumption Activates Astrocytes and Microglia and Exacerbates Neuroinflammation in Mouse Brains

**Figure 4:**
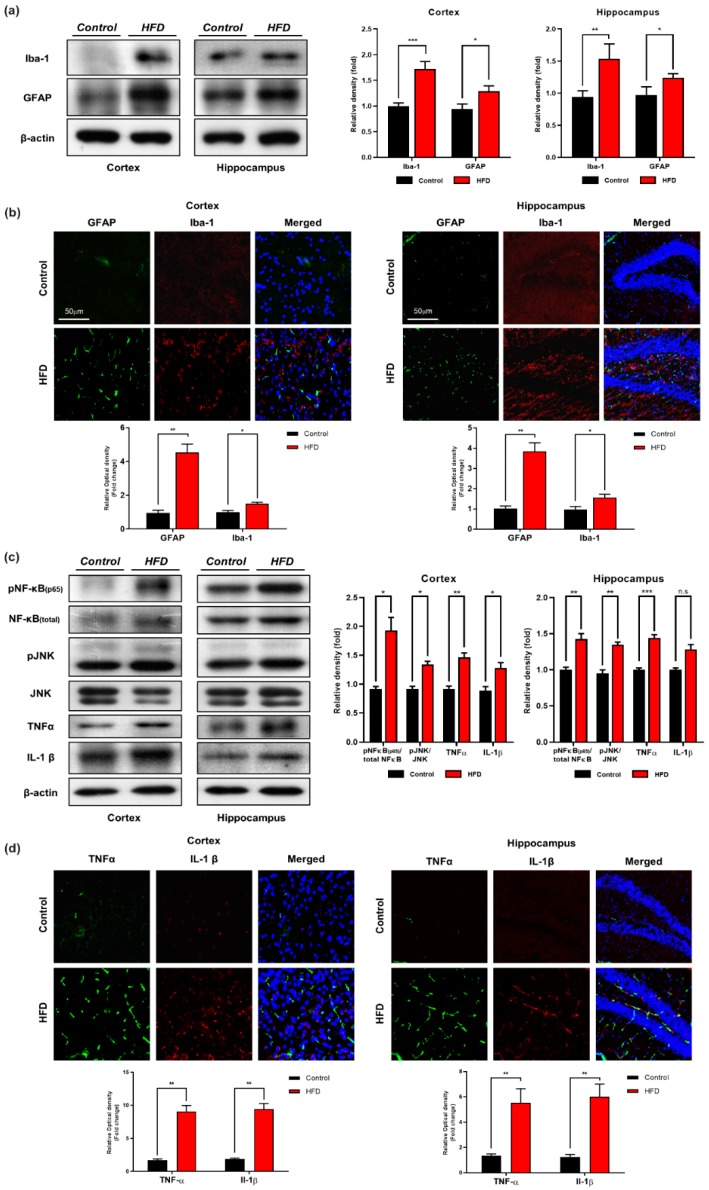
HFD increased level of activated microglia, astrocytes, neuroinflammatory markers and stress kinase -c-Jun N-terminal kinase (JNK)levels in HFD mouse brain. (**a**) Immunoblot result of ionized calcium binding adaptor molecule (Iba-1) and glial fibrillary acidic proteins (GFAP) in cortex and hippocampus (**b**) Immunofluorescence Imaging of Iba-1 and GFAP in cortex and hippocampus (**c**) Western blot result of tumor necrosis factor-alpha (TNFα), nuclear factor kappa B (NFkB), JNK and interleukin 1 βeta (IL-1β) in cortex and hippocampus (**d**) Confocal imaging with TNFα and IL-1β in the cortex and hippocampus of control and HFD group. Data are presented as mean ± SEM. Data for immunofluorescence and western experiment are compared using the Students *t*-test and other data are compared using one-way ANOVAs. Data are compared using U = the unpaired Students *t*-test. Significance = * *p* < 0.05, ** *p* < 0.01, *** *p* < 0.001. n.s = non-Significance.

Obesity caused inflammation (peripheral and central cytokines activation) have been linked with aberrant activation of c-Jun N-terminal kinase (JNK) [27]. Activation of glial cells (astrocytes and microglia) in response to HFD are key players in neuroinflammation [53]. We performed Western blot and confocal microscopic analyses of glial cells and neuroinflammatory markers in both the cortex and hippocampus of the mice that were fed the HFD for 24 weeks. An immunoblot and immunofluorescence analysis revealed that the affected regions of the HFD-fed mouse brains showed increased GFAP and Iba-1 expression and immunoreactivity, respectively, in both the indicated regions compared with those of the NCD-fed mouse brains (Figure 4a,b). Similarly, we also measured the expression of neuroinflammatory markers [p-NF-kB(65), IL-1β, TNFα] and JNK level in the brain regions of both HFD- and NCD-fed mice. Our Western blot analysis indicates that the consumption of the HFD significantly increased the expression of p-NF-kB(65), TNF-α, and IL-1β followed by the upregulated level of JNK in both the cortex and hippocampus regions compared with the levels observed in the NCD-fed mouse brains (Figure 4c). To further evaluate these results, we detected the pro-inflammatory cytokines IL-1β and TNFα by confocal microscopy. Consistent with our Western blot findings, higher immunoreactivity of pro-inflammatory cytokines (IL-1β and TNFα) was detected in both the cortex and hippocampus regions of mice fed the HFD compared with those of mice fed the NCD (Figure 4d). These findings indicate that chronic consumption of HFD initiates pro-inflammatory responses in two central nervous system (CNS) regions (cortex and hippocampus) in comparison with control-mice brains of the same age.

### 3.4. HFD Induces AD-Like Pathology in Mouse Brains

**Figure 5:**
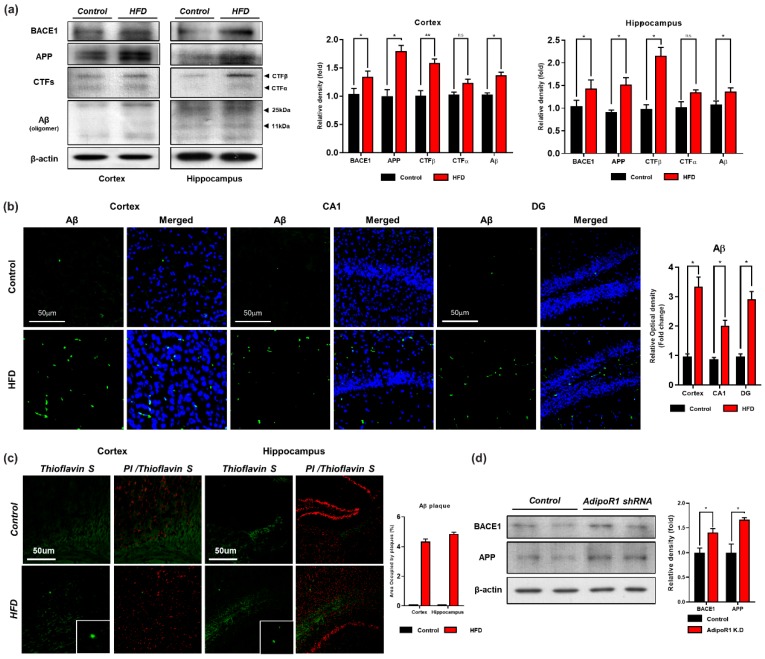
Chronic HFD up-regulates the amyloidogenic pathway in HFD mouse brain. (**a**) Represents the expression level of beta-amyloid-cleaving enzyme (BACE-1), amyloid precursor protein (APP), APP-C-terminal fragment βeta (APP-CTFβ) and amyloid beta (Aβ) proteins via western blot. β-actin was used as a loading control. (**b**) Given are the representative images of Immunofluorescence staining of Aβ in the cortex and hippocampal (CA1 and DG region) of the HFD mice brain (n = 12 mice/group) (**c**) Thioflavin S staining representing the sign of early plaque deposition in the cortex and hippocampus region of control and HFD mice brain. (**d**) Western blot analysis indicating expression level of BACE-1 and APP proteins in control and AdipoR1 knockdown in embryonic mouse hippocampal cell line mHippoE-14 (AdipoR1 shRNA). Data are presented as mean ± SEM. Data for immunofluorescence and western experiment are compared using the Student’s *t*-test and other data are compared using one-way ANOVAs. Data are compared using U\the unpaired Student’s *t*-test. Significance = * *p* < 0.05, ** *p* < 0.01. n.s= non-Significance.

Mounting evidence suggests that obesity, diabetes, and AD are linked through the disruption of neuronal insulin signaling [54,55]. In AD, APP is enzymatically cleaved by β-secretase (BACE) and then by γ-secretase, leading to Aβ production. Senile plaques and neurofibrillary tangles (NFTs) are two well-known and extensively studied hallmarks of AD that decrease synaptic plasticity [56]. Therefore, Western blot and confocal microscopy analyses of the cortex and hippocampus of both groups of mice were performed to assess the effect of the HFD on amyloid pathology. Our western blot results indicated that the expression level of APP and APP-CTFβ (APP C-terminal fragment indicating β-site cleavage of APP) was upregulated both in vivo and in vitro (Figure 5a,d). Consistent with these findings, we found that the expression level of BACE1 was increased in the HFD group compared with the control group (Figure 5a). In addition, our western blot results also indicated that knockdown of AdipoR1 in an embryonic mouse hippocampal cell line mHippoE-14 increased BACE1 level (Figure 5d). Moreover, we found increased expression and immunoreactivity of Aβ as detected in the brains of the HFD-fed mice via both immune blot and immunofluorescence, indicating that the consumption of an HFD induces signs of amyloid pathology (Figure 5a,b). Likewise, we examined the brains of both the NCD- and HFD-fed mice through thioflavin staining. The results indicated that the HFD significantly increased Aβ aggregation and deposition in the mouse brain in both indicated regions (cortex and hippocampus) and induced early signs of amyloid pathology, as evident from the minor accumulation of deposited amyloid senile plaques (SPs) (Figure 5b,c). Notably, our findings demonstrate that chronic consumption of HFD promotes BACE1-mediated APP cleavage and Aβ peptide generation and aggregation, resulting in signs of Aβ-containing SP deposition in the early stage of AD.

### 3.5. HFD Induces Synaptic Dysfunction and Memory Impairment in Mouse Brains

**Figure 6:**
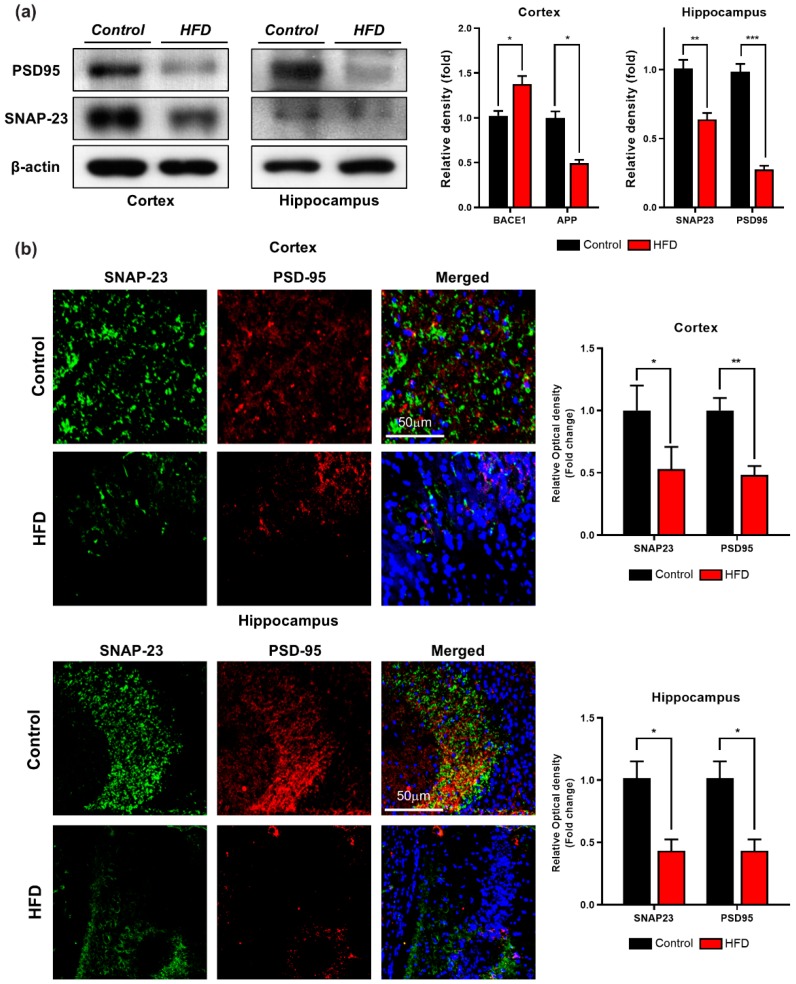
Effect of HFD on synaptic proteins in 24 weeks old HFD mice brain. (**a**) Western blot analysis of synaptosome-associated protein 23 (SNAP-23) and postsynaptic density protein 95 (PSD95) both in cortex and hippocampus along with their respective histograms. β-actin was used as a loading control. (**b**) Immunofluorescence images of pre and post synaptic proteins in the cortex and hippocampus of control and HFD mice brain. Data are presented as mean ± SEM. Data for immunofluorescence and western experiment are compared using the Student’s *t*-test, and other data are compared using one-way ANOVAs. Data are compared using the unpaired Student *t*-test. Significance = * *p* < 0.05, ** *p* < 0.01, *** *p* < 0.001.

Several studies have indicated that HFD has a substantial effect on a postsynaptic marker (drebrin, PSD-95) [57] and presynaptic proteins (synaptophysin, syntaxin, and SNAP-25) [14], leading to hippocampal long-term potentiation impairment [56,57,58]. Therefore, to determine the effect of HFD-induced synaptic loss, we investigated the pre- and postsynaptic protein levels in both groups through Western blotting analysis. As depicted in Figure 6a, the expression level of PSD-95 was significantly decreased in both the cortex and hippocampus of the HFD-fed group in comparison with the control group. A presynaptic protein marker (synaptosomal-associated protein 23 (SNAP-23) that plays a vital role in synaptic plasticity [59] was also analyzed through Western blot analysis.

Consistent with the previous findings, the expression of (SNAP-23) in both the cortex and hippocampus was decreased in the HFD-fed group in comparison with the control group (Figure 6a). To further strengthen these results, we performed immunofluorescence staining of the before-mentioned synaptic protein markers, and our immunofluorescence staining results revealed that the immunoreactivity of pre- and postsynaptic markers (SNAP-23 and PSD-95, respectively) was significantly reduced in both brain regions of the HFD-fed mice in comparison with the control group (Figure 6b). These findings indicate that HFD decreases the expression of both pre- and postsynaptic proteins in the affected regions of the mouse brain, leading to impairments in synaptic deregulation.

**Figure 7:**
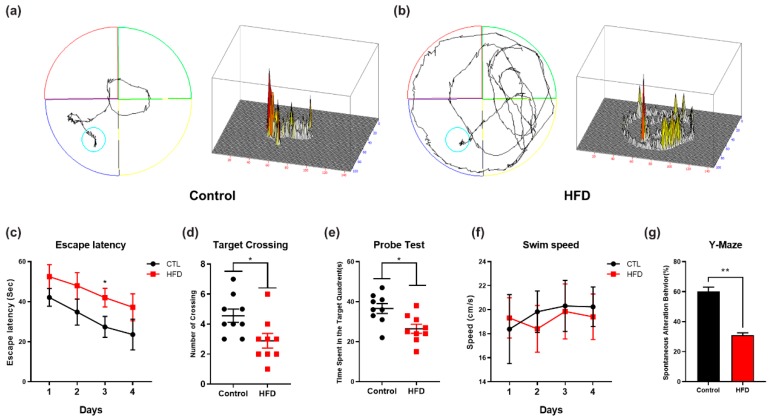
Effect of HFD on spatial learning, memory, and cognitive impairment (**a**,**b**) Representative maps indicates trajectories path lengths covered by the mice during probe test, in the Morris water maze (MWM) test (**c**) Mean escape latency (sec) indicated by line graph to reach the hidden platform during the training sessions, in the MWM test (**d**,**e**) Target crossing or number of platform crossing and time spent in the target quadrant during the probe test, in the MWM test (**f**) Line graph representing swimming speed (cm/s) of the mice during their search for the hidden platform during the training sessions before the probe test. To calculate swimming speed (cm/s), we used the equation v = distance/time. No significant difference was observed between the swimming speeds of the experimental groups. (**g**) The histogram analysis of percentage spontaneous alternation in the Y-Maze test, * *p* < 0.05, using Student’s *t*-test against their respective normal chow diet control. Significance = * *p* < 0.05, ** *p* < 0.01.

To evaluate the impact of the HFD on cognitive performance, we performed behavioral analyses, including the MWM and Y-maze tests. The MWM test is the most reliable and noninvasive test for determining cognitive changes in the AD mouse model [60]. Accordingly, we used the MWM and Y-maze tests to assess the effect of the HFD on memory impairment. First, we recorded the learning ability of the mice (n = 12 mice/group) during the training session in the MWM test. We observed that the HFD-fed mice showed more latency time in seconds to reach the hidden platform in comparison with control mice (Figure 7c). We also examined the mean swim speeds during the training days in order to observe the motor ability between HFD-fed mice and NCD-fed mice. We found no significant difference in swim speed measurements between HFD-fed mice and NCD-fed mice, indicating that any differences in ability to reach the hidden platform were irrelevant to the motor functions of the mice (Figure 7f). Next, after completion of the training session, we removed the hidden platform, and performed a probe test. The results of the probe test showed that the control mice fed with NCD spent more time in the target quadrant and exhibited a higher number of platform crossings, whereas the HFD-fed mice not only spent less time in the target quadrant but also exhibited few platform crossings (Figure 7d,e). This finding shows that the chronic consumption of HFD induces memory impairment. Following the MWM test, we evaluated the spontaneous alteration behavior percentage of the mice (n = 12 mice/group) by evaluating the average total number of arm entries and successive triplets using a Y-maze test. The spontaneous alternation behavior provides an indication of spatial working memory, which is a type of short-term memory. After the feeding period, the percentage of spontaneous alternation behavior of the HFD-fed mice was lower than that of the control mice, which suggests that the HFD is responsible for the observed decline in cognition (Figure 7g).

## 4. Discussion

Metabolic syndrome, which includes obesity and diabetes, plays a pivotal role in AD pathophysiology [55]. Insulin resistance might play an important role in the development and progression of AD. High-fat diets are associated with obesity, type 2 diabetes, and IR [61,62]. Although the effect of dietary manipulation on cognitive functions has been studied, the effect of the chronic consumption of n HFD on AD-like pathology has not been examined. In the present study, we assessed the effect of an obesogenic diet and metabolic-stress-associated oxidative stress, neuroinflammation, suppression of the AdipoR1-mediated signaling pathway, amyloid pathology and cognitive function through a comparative analysis of the brains of NCD-fed mice and HFD-fed mice. Therefore, to induce obesity (i.e., an obese state), we fed male mice with HFD for 24 weeks. Our findings indicated that the consumption of HFD triggered metabolic perturbations, leading to alterations of the antioxidant system through suppression of the AdipoR1 signaling pathway that leads to neuronal insulin resistance in mouse brains. To strengthen our results, AdipoR1 knockdown in embryonic mouse hippocampal cell line mHippoE-14 also showed increased oxidative stress. Next, we also found that HFD leads to neuroinflammation. Furthermore, the mice fed with HFD showed synaptic dysfunction and induced impairments in hippocampal-dependent spatial learning and memory as accessed by behavioral analysis. Taken together, these results showed the development of central insulin resistance in the HFD-fed mouse brains, as demonstrated by the attenuation of AdipoR1/AMPK activation, which may be the possible mechanism of Aβ deposition and the early onset of AD pathogenesis.

Obesity and type 2 diabetes are linked to a chronic inflammatory response in multiple tissues, including adipose and brain tissue [63,64]. In the periphery, adipose tissue-derived pro-inflammatory and inflammatory mediators produced by activated resident brain inflammatory cells (astrocytes and microglia) enhance central inflammation in obesity [25,27]. Glial cells, including astrocytes and microglia, are by far the most abundant cells in the mammalian CNS having multiple roles [65]. For example, previous studies have demonstrated that HFD-consumption-induced obesity causes CNS inflammation, followed by astrogliosis [66] and microgliosis [67] in rodents, which can be recognized by elevations in their respective markers, including GFAP (an astrocytic marker) and Iba-1 (microglia marker) [68]. Therefore, consistent with these previous findings, we determined the effects of HFD feeding on glial activation. Our Western blot and immunofluorescence analyses indicated significant increases in expression and immunoreactivity in GFAP and Iba-1 levels in both the cortex and hippocampus of HFD-fed mice brains in comparison with the NCD-fed mice brains. Moreover, other studies reported that TNF-α is overexpressed in the adipose tissue of obese individuals [28], and this cytokine activates neuronal cytokine receptors (e.g., TNF-α receptor) that cause aberrant activation of stress-sensitive kinases (c-Jun N-terminal kinase; JNK, IkBα kinase; IKK and double-stranded RNA-dependent protein kinase; PKR), leading to insulin resistance [27,29]. Therefore, in obesity, both adipose-tissue-derived pro-inflammatory mediators and cytokines produced by CNS-resident microglia exacerbate neuronal inflammation [26], which is a common feature of AD [69]. Accordingly, our immunoblot and immunofluorescence results also indicated that HFD leads to activation of stress kinase (JNK) and neuroinflammatory markers (TNFα and IL-1 β) in HFD-fed mouse brain (Figure 4c,d). Together, these data suggest that the chronic consumption of HFD (24 weeks) can lead to significant brain inflammation and strongly argue that an appropriate diet is crucial for cognitive health.

Insulin signaling in the brain reportedly regulates key processes, including neuronal growth, synaptic plasticity, and energy homeostasis [70], and the disruption of insulin signaling is recognized as an important risk factor for AD pathogenesis [50,71]. Noticeably, APN has been reported to have insulin-sensitizing and anti-inflammatory actions [72] by activating multiple signaling molecules. Of note, AMPK is a major downstream molecule of APN signaling. In general, AMPK acts as the cellular energy or fuel sensor responsible for the maintenance of cellular energy homeostasis in the body and is normally activated in response to an increase in the intracellular AMP/ATP ratio [73,74,75], e.g., the depletion of cellular energy by stress, starvation, hypoxia, or other means causes an increase in intracellular AMP and thereby, allosterically activates AMPK through phosphorylation of its α-subunit at Thr-172 by upstream kinases [76]. Reduced circulating APN levels have been observed in aged obese individuals and obese patients with type 2 diabetes mellitus [77,78]. Importantly, chronic APN deficiency due to AMPK inactivation causes cerebral insulin resistance in mice, i.e., increases IRS-1 phosphorylation at serine 616 and inhibits the formation of pIRS-1Tyr in neurons, leading AD-like pathologies [46]. Similarly, impaired brain insulin signaling is also associated with pro-inflammatory signaling: abnormal serine phosphorylation of IRS-1 (IRS-1pSer636) is caused by TNF-α activation via the JNK/TNF-α pathway and is blocked by infliximab, a TNF-α neutralizing antibody [79]. Furthermore, previous studies have revealed that HFD constitutes a well-established model for the induction of insulin resistance in both the periphery and brain [57] because the brain of HFD-fed mice show increased IRS-1 phosphorylation at Ser307 and inhibited IRS-1 phosphorylation at Tyr608 in comparison with the brains of control mice [80]. Consistent with these findings, we further elucidated that the consumption of the HFD is associated with disruption of the critical insulin signaling pathway with a particular focus on the adipoR1/AMPK/IRS-1 pathway. Our results revealed that the expression of adipoR1 and p-AMPK was reduced in both the cortex and the hippocampus region of the HFD-fed mouse brains. Noticeably, the HFD, through AMPK inactivation, increased the phosphorylation of IRS-1 at serine 616 and thus prevented its interaction with the insulin receptor (InsR) and downstream signaling molecules, leading to the mislocalization of IRS and thereby, inhibiting insulin signaling, as evident from the reduced expression of pPI3K and p-Akt (Ser473). To our knowledge, our findings provide the first indication that chronic consumption of an HFD increases central insulin resistance through the AdipoR1/AMK/IRS-1 pathway in both the cortex and hippocampus of the mouse brain and, ultimately, leads to Aβ accumulation, resulting in AD-like neuropathy.

The production and accumulation of excess Aβ, a primary cause of the onset and progression of AD, are induced by the sequential cleavage of APP by β- and γ-secretases. The formation of amyloid plaques composed of Aβ fragment is considered a characteristic hallmark of AD pathology [81]. Accumulative lines of evidence strongly indicate that HFD enhances the cleavage of APP by accelerating BACE-1 activity, which exacerbated Aβ pathology [58,81,82]. Accordingly, we investigated the effect of the HFD on amyloid pathology to ascertain whether the HFD influences the processing of APP and the accumulation of Aβ peptides. We analyzed the APP, BACE-1, and Aβ levels in the brains of HFD-fed and control mice and found that the expression of BACE-1 was increased in both the cortex and hippocampus region of the brains of HFD-fed mice in comparison with the NCD-fed mice (Figure 5a). Thus, an HFD accelerates the β-secretase-mediated processing of APP to Aβ. We also considered the possibility that the memory impairment detected in HFD-fed mice might be due to ample Aβ deposition, and we thus analyzed Aβ accumulation in both regions of the mouse brains. The results showed that Aβ deposition was aggravated in the cortex and hippocampus of the HFD-fed mice brains in comparison with the control mice brains. Furthermore, thioflavin S staining showed Aβ plaque deposition in the cortex and hippocampus in the brains of HFD-fed mice in comparison with the control mice. These data suggest that the consumption of an obesogenic diet, such as an HFD, for 24 weeks, accelerates the BACE1-mediated cleavage of APP, which results in Aβ production followed by amyloid plaque deposition.

**Figure 8:**
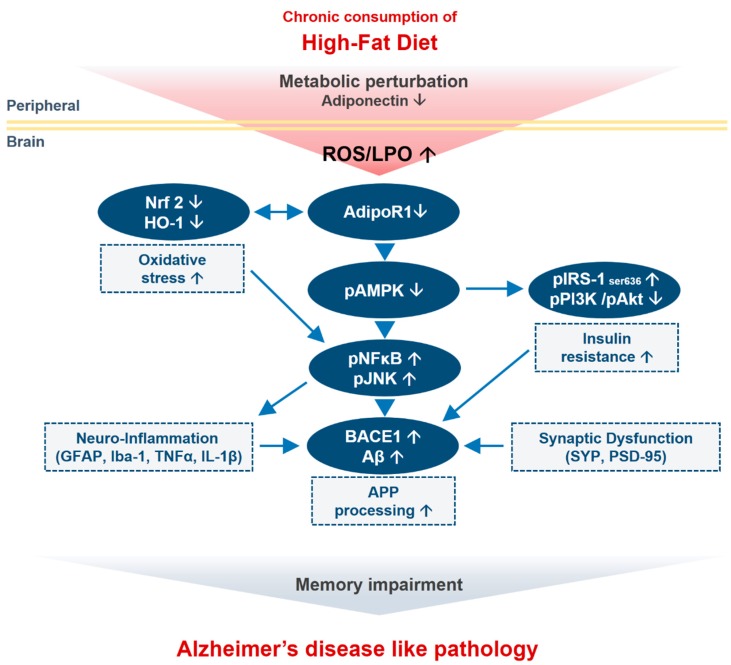
Schematic outline representing neuronal adiponectin signaling pathway in the brain upon consumption of HFD. Chronic consumption of HFD along with several inciting factors (metabolic perturbation and adiponectin deficiency) in periphery leads to oxidative stress and AdipoR1 suppression along with its downstream signaling molecules (energy depletion; p-AMPK) in the brain. This leads to insulin resistance and increased APP processing (increased in BACE-1 and Aβ level). Abnormal accumulation of Aβ is accompanied by neuroinflammation and synaptic dysfunction that together leads to memory impairment and Alzheimer-like pathology in the mouse brain.

Synaptic loss is believed to form the basis of cognitive impairment in the early phase of AD [83] due to reduced expression of synaptic proteins [84]. Previous studies revealed that excessive dietary intake reduces the expression of synaptic markers, including presynaptic vesicle marker (SYP) and a postsynaptic density protein (PSD-95), in an animal model of obesity [58,85]. Accordingly, our immunoblot and immunofluorescence findings also indicated a significant decrease in the expression and immunoreactivity of synaptic proteins (PSD-95 and SNAP-23) in the cortex and hippocampus regions of HFD-fed mice compared with NCD-fed mice. Similarly, several lines of evidence have reported that mice with chronic APN deficiency and mice fed with HFD shows behavioral deficits, including spatial memory and learning impairments [50]. Therefore, in addition to significant synaptic loss, behavior deficits have also been detected after the chronic consumption of HFD. The MWM test results showed that the HFD-fed mice exhibited longer escape latencies, reduced swimming times in the target quadrant, reduced number of platform crossings, and less time spent in the target quadrant compared with the control-diet-fed group. These results indicated that HFD could lower the learning and memory abilities of mice. In summary, our study supports the notion that the chronic consumption of HFD induces cognitive impairments through the adipoR1/AMPK/IRS1 axis, leading to the development of AD-like pathology (Figure 8).

## 5. Conclusions

The majority of studies investigating variable diets, aging models, and behavioral analysis support the notion that exposure to HFD has a notable and long-lasting impact on learning and memory. The consumption of HFD accelerates cognitive dysfunction, neuronal insulin resistance, neuroinflammation, and loss of synapse density, leading to amyloid pathology in the brain. Herein, our results indicated that HFD induces biochemical or metabolic perturbations that elevated neuronal oxidative stress and brain insulin resistance through AdipoR1 suppression in HFD-fed mice. Notably, these findings are supported by our in vitro results, in which knockdown of AdipoR1 in an embryonic mouse hippocampal cell line mHippoE-14 leads to increased oxidative stress in neurons. Moreover, the chronic consumption of HFD leads from neuroinflammation to impaired memory function, synaptic loss through the AdipoR1/AMPK pathway by inhibiting IRS-1 at serine 616 and its downstream PI3-K/Akt pathway. Importantly, our data suggest that a loss of AMPK activation through AdipoR1 might constitute a potential mechanism through which cells develop neuronal insulin resistance, leading to a decline in cognitive function. Furthermore, our findings provide convincing evidence showing that HFD-induced metabolic dysfunctions lead to Aβ deposition and memory impairments through the adipoR1/AMPK/IRS1 axis, leading to the development of AD-like pathologies. Future pre-clinical efforts will be helpful for establishing the exact molecular mechanisms involving the effects of metabolic manipulations on the pathogenesis of neurodegeneration.

## Supplementary Materials

The supplementary materials are available online at https://www.mdpi.com/2073-4409/9/1/249/s1.
