# Supplementary material for: Metabolic Stress Alters Antioxidant Systems, Suppresses the Adiponectin Receptor 1 and Induces Alzheimer’s Like Pathology in Mice Brain
Source: Cells. 2020 Jan 19;9(1):249. doi: 10.3390/cells9010249 (PMC7016950; doi:10.3390/cells9010249)
Supplement: Supplementary file 1 [file cells-09-00249-s001.pdf]

Supplementary Figure

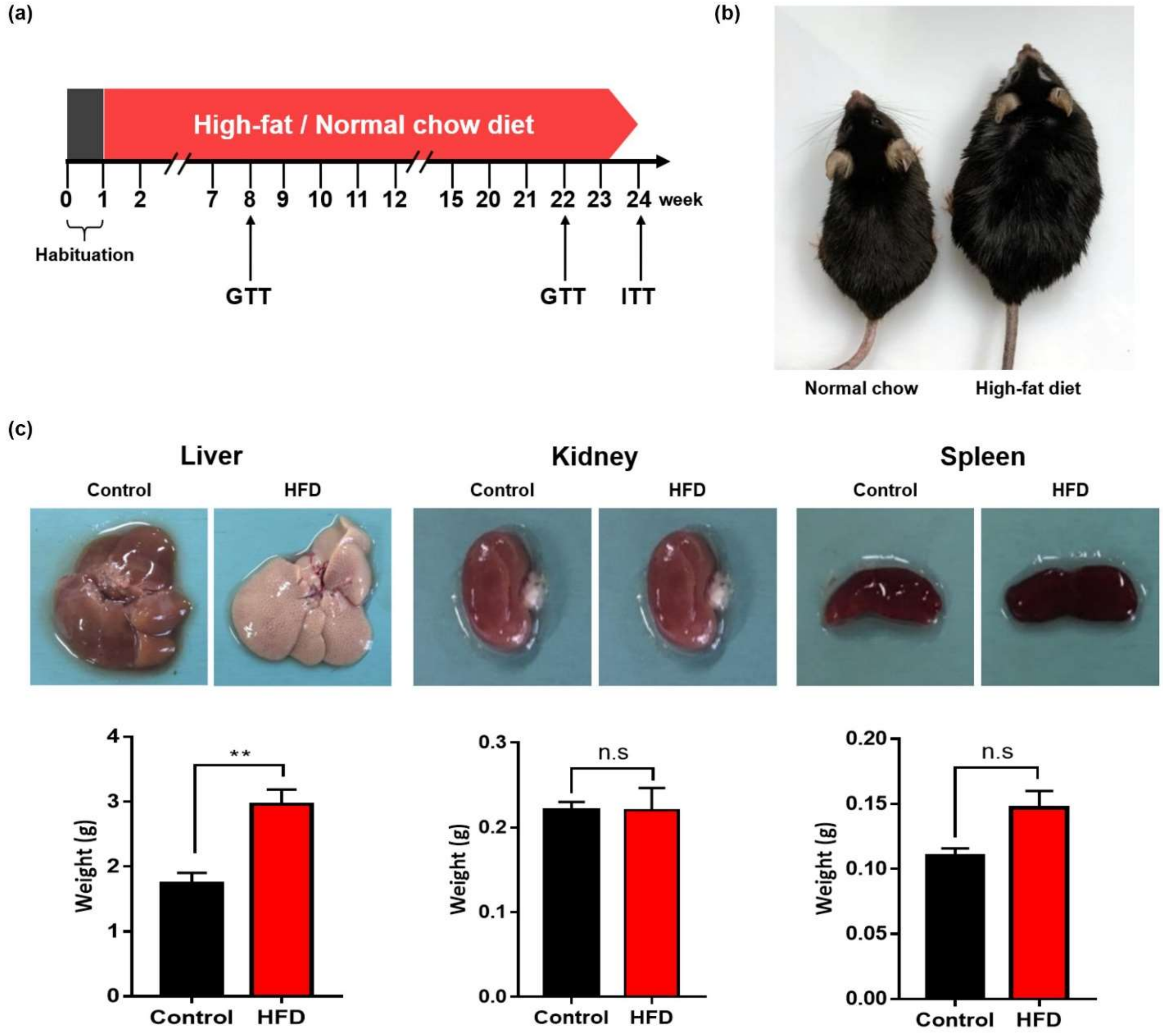

Supplementary Figure. Effects of High Fat Diet on Peripheral Organs Weight. (A) Represents the HFD modelling scheme (B) Phenotypic representation of wild type and HFD induce mice (C) Representative images and their respective histograms of peripheral organs weight. Data are compared using Unpaired Student t-test. *$p < 0.05$.
